# Pyrolysis Kinetics and Combustion Behaviors of a High-Nitrogen Compound, 4,4′-Azobis(1,2,4-triazole)

**DOI:** 10.3390/ijms231911313

**Published:** 2022-09-25

**Authors:** Qi Pan, Honglei Zhang, Xueyong Guo, Sen Sun, Shenghua Li

**Affiliations:** 1 State Key Laboratory of Explosion of Science and Technology, Beijing Institute of Technology, Beijing 100081, China; 2 Beijing Institute of Electronic System Engineering, Beijing 100854, China; 3 School of Materials Science and Engineering, Beijing Institute of Technology, Beijing 100081, China

**Keywords:** ATRZ, thermal analysis, thermal properties, combustion

## Abstract

To study the thermal decomposition behavior of 4,4′-azobis(1,2,4-triazole) (ATRZ), the non-isothermal thermal decomposition kinetics of ATRZ were studied using the thermogravimetric–differential scanning calorimetry (TG–DSC) method. The TG–DSC of ATRZ was analyzed at heating rates of 5, 10, 15, and 20 K·min^−1^ in an argon atmosphere. The thermal decomposition kinetic parameters at peak temperature (*T_p_*), such as apparent activation energy (*E_a_*) and pre-exponential factor (lg*A*) of ATRZ, were calculated using the Kissinger, Ozawa, and Satava–Sestak methods. *E_a_* and lg*A* calculated using the Kissinger, Ozawa, and Satava–Sestak methods are very close, at 780.2 kJ·mol^−1^/70.5 s^−1^, 751.1 kJ·mol^−1^/71.8 s^−1^, and 762.1 kJ·mol^−1^/71.8 s^−1^, respectively. Using a combination of three methods, the reaction mechanism function g(α) of ATRZ was obtained. The results show that the decomposition temperature of ATRZ is about 310 °C, and the decomposition is rapidly exothermic. The pyrolysis path of ATRZ was investigated through a pyrolysis-gas chromatography mass spectrometry (PY-GC/MS) experiment. ATRZ has three different decomposition paths and finally generates N_2_, HC-N-CH, N≡C-N, and HC=N-C≡N. The laser ignition combustion duration of ATRZ was 0.5033 s and the peak temperature was 1913 °C. The laser ignition combustion duration of ATRZ+CL-20 was 1.0277 s and the peak temperature was 2105 °C. The rapid energy release rate of ATRZ promotes the combustion energy release of CL-20.

## 1. Introduction

In recent years, more and more attention has been paid to the theoretical calculation, synthesis, and application of polynitrogen compounds [1,2,3,4], especially nitrogen-rich heterocyclic-based energetic compounds. Nitrogen-rich heterocyclic-based energetic compounds such as tetrazole, triazole, pyrazole, imidazole, and oxadiazole are very promising candidates [5,6]. Singly or doubly bonded polynitrogen compounds can decompose into dinitrogen (N_2_), with an extremely large and rapid energy release, which makes them attractive as potential explosives or propellants [7,8,9]. Single- and double-bond nitrogen systems have a higher heat of formation. It has also been found that the heat of formation of systems with continuous nitrogen atoms is higher than that of cyclic polynitrogen compounds with a discontinuous nitrogen distribution [10]. 4,4′-azobis(1,2,4-triazole) (ATRZ) is a polynitrogen compound with a high nitrogen content [11]. Related research work has been carried out on ATRZ, including molecular dynamics calculations of its thermal decomposition properties, while thermodynamic methods have been used to study its thermal decomposition properties and the use of ATRZ as a high-energy-density metal–organic framework [12,13,14,15]. In addition, nitrogen-rich salts based on polyamino-substituted N,N′-azo-1,2,4-triazole are also a new family of high-performance energetic materials [16]. The thermal decomposition behavior of explosives, including their kinetics, mechanisms, and interactions with additives, has attracted much attention, since it directly determines the thermal stability of explosive-based composite energetic materials when exposed to external stimuli [17,18,19]. Combustion is a type of reaction with a faster rate of energy release than thermal decomposition. The thermal analysis and combustion behaviors of new polynitrogen compounds is of great significance for the qualitative description of their reaction laws in the combustion process, the establishment of mathematical models, the calculation of their kinetic and thermodynamic parameters, and the development and application of new polynitrogen compounds.

The weight percentage of N element in the ATRZ molecule is 68.29%, and the molecule contains C-N bonds, N-N bonds, and N=N bonds, which have high theoretical energy storage. The crystal morphology and element distribution of ATRZ have been characterized. To study the thermal stability of ATRZ, the non-isothermal thermal decomposition process of ATRZ was analyzed through thermogravimetric (TG) analysis and differential scanning calorimetry (DSC) in this study. The activation energy (*E_a_*) and pre-exponential factor (lg*A*) of its thermal decomposition reaction process were obtained, laying a foundation for its application in explosives. In order to explore the reaction mechanism of ATRZ in a rapidly heating environment, the thermal pyrolysis process of ATRZ was investigated using pyrolysis-gas chromatography mass spectrometry (PY-GC/MS). The combustion duration and temperature of ATRZ were studied through laser ignition. The effects of the rapid energy release property of ATRZ on the combustion of 2,4,6,8,10,12-hexanitro-2,4,6,8,10,12-hexaazaisowurtzitane (CL-20) were investigated.

## 2. Results and Discussion

### 2.1. Morphology Characterization of ATRZ

**Figure 1:**
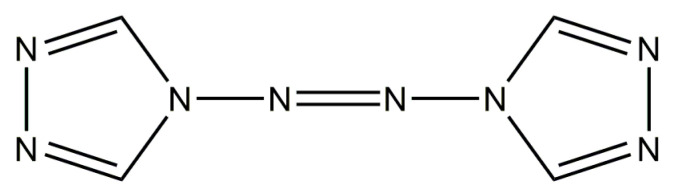
Molecular structure of ATRZ.

ATRZ is composed of two triazole rings and azo bonds and has a symmetric coplanar molecular structure. Its molecular structure is shown in Figure 1. ATRZ has an excellent nitrogen content, with a theoretical content of 68.29% and a carbon content of 29.27%. The ATRZ molecule contains one N=N double bond, four N-N single bonds, and four C=N double bonds, which have a high energy storage in theory. Additionally, the molecule does not contain the nitro group, which has a better safety profile.

**Figure 2:**
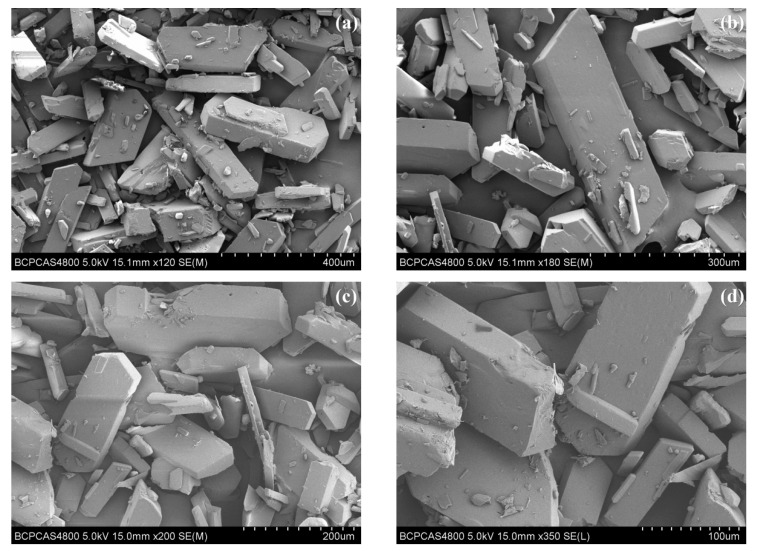
Crystal morphology of ATRZ characterized by SEM. 400 μm (**a**), 300 μm (**b**), 200 μm (**c**), and 100 μm (**d**).

The crystal morphology of ATRZ is shown in Figure 2a–d. The crystal morphology of ATRZ features an irregular block structure, and its length is roughly 150–300 μm. The long to short axis ratio of ATRZ crystal is about 2.0. There are no obvious crystal defects on the surface of ATRZ crystal. Within the visual range, it can be seen that about 80% are large-sized crystals of ATRZ, while the rest are crystal debris of ATRZ.

**Figure 3:**
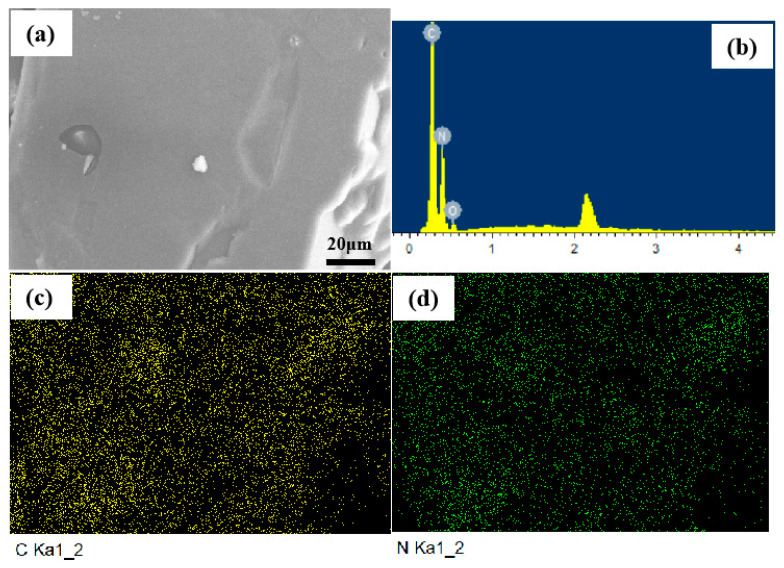
Elemental distribution of ATRZ. SEM image of ATRZ (**a**), the ratio of C and N elements (**b**), C element distribution (**c**) and N element distribution (**d**).

The part shown in Figure 3a was selected for element distribution analysis. Figure 3b–d show the surface element distributions of ATRZ. The mass percentage of N element was 65.24% and the mass percentage of C element was 33.13%, which is in agreement with the theoretical calculation value.

### 2.2. Thermal Decomposition Kinetics of ATRZ

**Figure 4:**
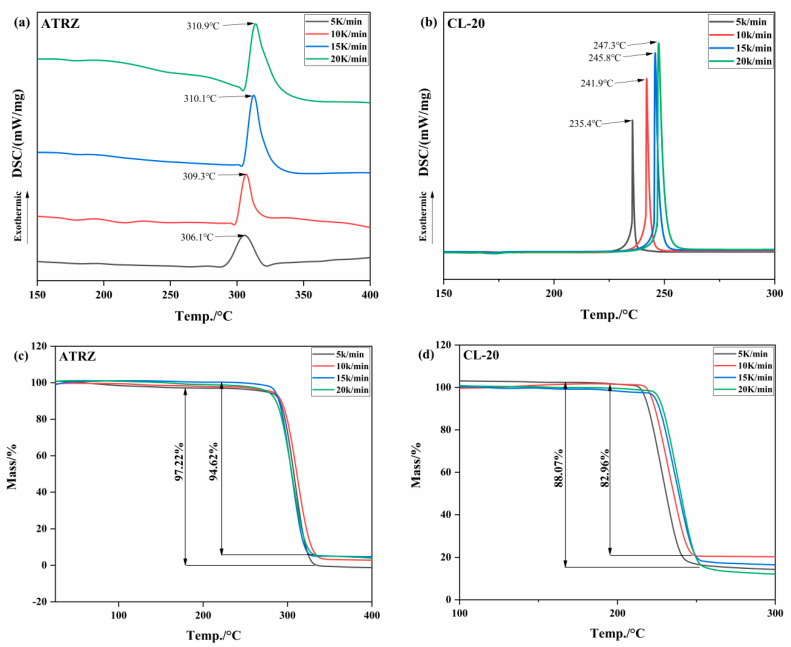
TG–DSC curves of ATRZ and CL-20 at different heating rates. DSC curve of ATRZ (**a**), DSC curve of CL-20 (**b**), TG curve of ATRZ (**c**) and TG curve of CL-20 (**d**).

The heat flow and thermo-gravimetric curves of ATRZ and CL-20 at different heating rates are shown in Figure 4. The results show that the decompositions are exothermic processes at the experimental temperatures. With the increase in the heating rate, the thermal decomposition peak temperature (*T_p_*) of ATRZ gradually increased, the *T_p_* was stable at around 310 °C, and the *T_p_* of CL-20 was around 240 °C. At every heating rate, the *T_p_* of ATRZ was higher than that of CL-20. As shown in Figure 2c,d, the weight loss of ATRZ was between 94.62 and 97.22%, while the weight loss of CL-10 was between 82.96 and 88.07%. The thermal decomposition reaction of ATRZ involves the cleavage of its intermolecular N-N single bond and N=N double bond, and no intramolecular redox reaction occurs. The thermal decomposition of traditional ammonium nitrate explosives is mainly an intramolecular redox reaction, and the presence of nitro groups also greatly increases the sensitivity of CL-20.

The kinetic parameters of ATRZ and Cl-20 at *T_p_* were fitted and calculated by Kissinger’s Equation (1) [18].

(1) $$\ln\frac{\beta }{T_{p}{}^{2}}=\ln\frac{A_{K}R}{E_{K}}-\frac{E_{K}}{R}\frac{1}{T_{p}}$$

where *T* is the reaction thermodynamic temperature, *R* is the gas molar constant (8.314 J·mol^−1^·K^−1^), *E_K_* is the apparent activation energy, *A_K_* is the pre-exponential factor, and *β* is the heating rate.

**Figure 5:**
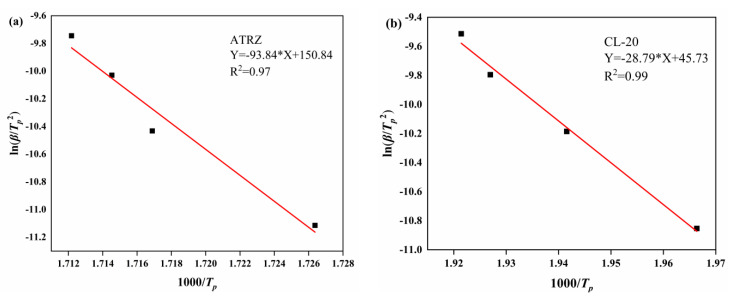
Kissinger method fitting curves. Kissinger method of ATRZ (**a**) and Kissinger method of CL-20 (**b**).

Figure 5 shows that the thermal decomposition kinetic curves of ATRZ and CL-20 fitted by the Kissinger method have a good degree of fit. The *T_p_* values at different heating rates and kinetics of ATRZ and CL-20 are listed in Table 1. From the original data shown in Table 1, the values of *E_K_* and lg*A_K_* obtained by the Kissinger method are listed. ATRZ had a higher *E_K_* than CL-20, indicating that ATRZ has a better thermal stability than CL-20 at different heating rates. Additionally, the lg*A_K_* of ATRZ was also greater than that of CL-20, indicating that ATRZ has a higher energy release rate. The main reason for this is that the energy release form of ATRZ is the breaking of the intramolecular N-N single bond and N=N double bond.

**Figure 6:**
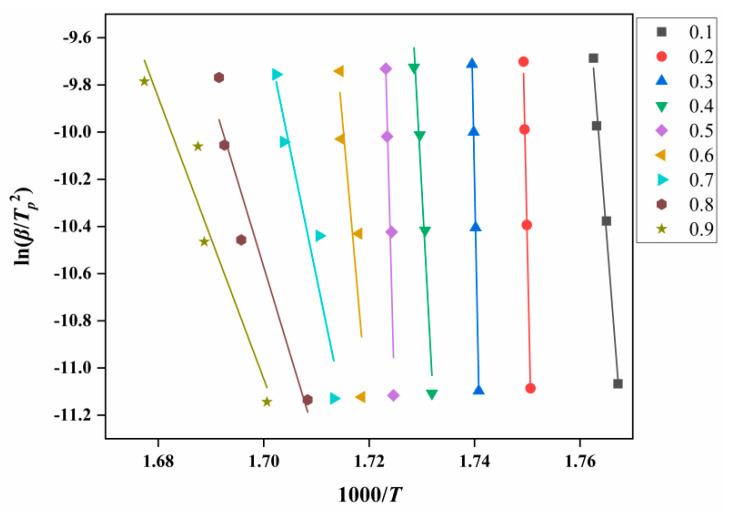
*E_k_* analysis diagram of ATRZ determined by the Kissinger method.

**Figure 7:**
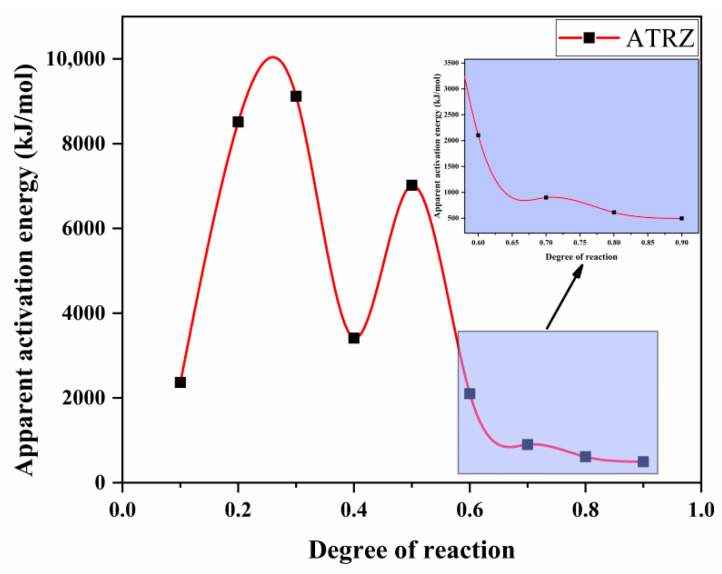
The *E_k_* curves of pyrolysis vs. degree of reaction determined by the Kissinger method.

According to Equation (1), a linear plot of ln(*β*/*T_p_*^2^) against 1000/*T* at the same fractional conversion was drawn, as shown in Figure 6. Figure 7 shows the variation in *E_K_* with the degrees of reaction determined by Kissinger. It can be seen from Figure 7 that the thermal decomposition process of ATRZ can be roughly divided into two stages. The first stage was before the reaction depth of 0.6. At this time, the *E_k_* was high, the decomposition was relatively slow, and it was in the endothermic stage. The second stage was the rapid decomposition stage, which released a lot of heat and produced a lot of gas. This was also verified by the TG–DSC curve. The reaction depth at the peak temperature was between 0.6 and 0.7, and the apparent activation energy at this time also corresponded.

The Ozawa method and the Satava-Sestak method were used to further study the thermal decomposition process of ATRZ. Ozawa’s Equation (2) and Satava-Sestak’s Equation (3) are as follows:

(2) $$\mathrm{lg}\beta =\mathrm{lg}[\frac{A_{o}E_{o}}{Rg(\alpha )}]-2.315-0.4567\frac{E_{o}}{RT_{}}$$

(3) $$\ln g(\alpha )=\ln\frac{A_{S}E_{S}}{R\beta }-5.330-1.0516\frac{E_{S}^{}}{RT}$$

Using the Satava–Sestak method, the 30 mechanism functions given in Table 2 were used to determine a linear relationship between ln*g*(*α*) and 1/*T*. Then, the apparent activation energy *E_S_* and pre-exponential factor *A_S_* were obtained from the slope. According to the calculation results, we selected the *A_S_* corresponding to the activation energy *E_S_* in the range of 0 < *E_S_* < 400 kJ·mol^−1^. Compared with the activation energy obtained by the Ozawa method, the obtained activation energy satisfied the condition |(*E_O_* − *E_S_*)/*E_O_*| ≤ 0.1. Additionally, compared with the ln*A_K_* obtained by the Kissinger method, the ln*A_S_* was in the form |(ln*A_S_* − ln*A_K_*)/ln*A_S_*| ≤ 0.46. Only the integral function *g*(*α*) satisfying both conditions can be the integral form of the reaction mechanism function of thermal decomposition.

**Figure 8:**
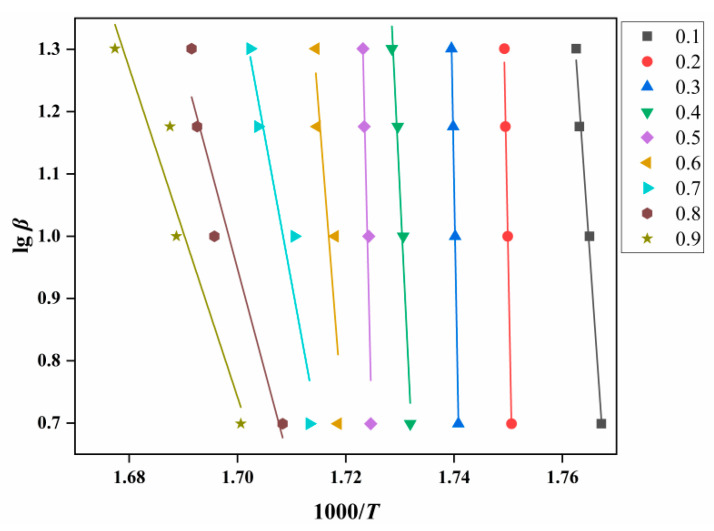
*E_O_* analysis diagram of ATRZ created by the Ozawa method.

**Figure 9:**
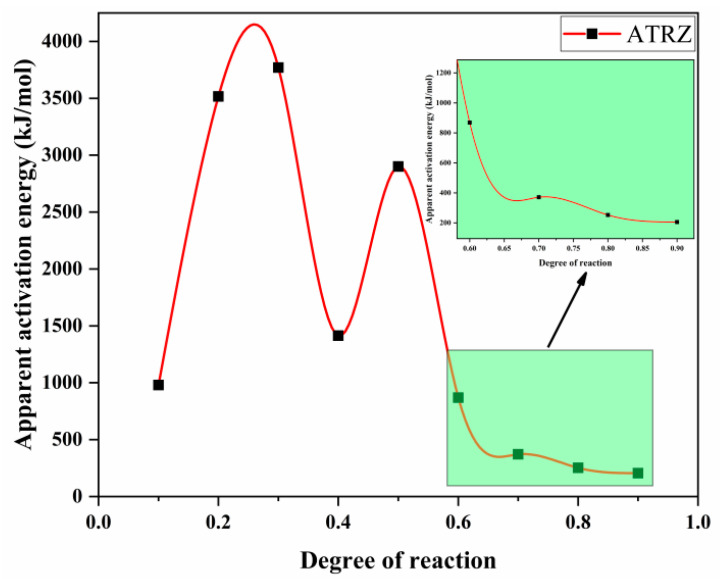
The *E_O_* curves of pyrolysis vs. degree of reaction created by the Ozawa method.

Through calculation and screening, No. 3 in Table 2 was obtained as the mechanism function corresponding to *g*(*α*). According to Equation (2), a linear plot of lg*β* against 1000/*T* at the same fractional conversion can be drawn as Figure 8. Figure 9 shows the variation in *E_O_* with the degrees of reaction by Ozawa.

(4) $$g(\alpha )=(1-\frac{2}{3}\alpha )-{\left(1-\alpha \right)}^{\frac{2}{3}}$$

According to the *g*(*α*) obtained by computational screening, the apparent activation energy and pre-exponential factor at the peak temperature were calculated using the Ozawa method and the Satava–Sestak method, respectively, as shown in Table 3. The apparent activation energy and the pre-exponential factor at the peak temperature calculated by the three methods were relatively close, and the calculated results were accurate.

### 2.3. Thermal Pyrolysis Analysis of ATRZ

**Figure 10:**
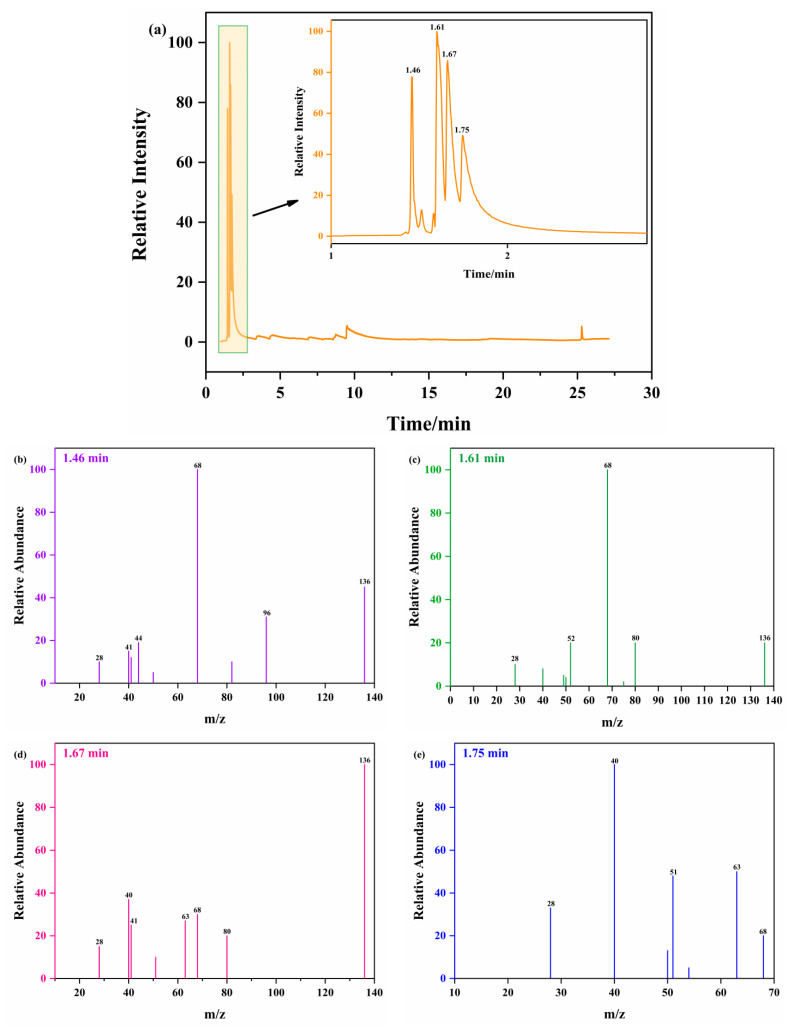
PY-GC/MS pyrolysis spectra of ATRZ at 400 °C (**a**) and MS spectra of ATRZ at 1.46 min (**b**), 1.61 min (**c**), 1.67 min (**d**), and 1.75 min (**e**).

PY-GC/MS was used to investigate the decomposition processes and pyrolysis products of ATRZ. The decomposition path and mechanism of ATRZ were analyzed. Figure 10a shows the total ion fragmentation chromatogram of ATRZ. ATRZ mainly showed chromatographic peaks at retention times of 1.46, 1.61, 1.67, and 1.75 min, with the most abundant being at 1.61 and 1.67 min. The mass spectra corresponding to the four chromatographic peaks are shown in Figure 10b–e, respectively.

**Figure 11:**
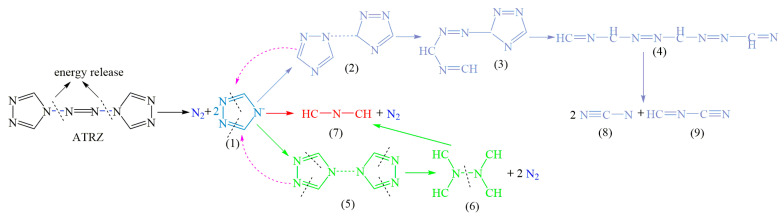
The concise pyrolysis pathway of ATRZ.

According to the total ion fragmentation chromatogram of ATRZ shown in Figure 10 and the corresponding mass spectra of the chromatographic peaks of the four main retention times, the pyrolysis path of ATRZ was inferred, as shown in Figure 11. ATRZ was initially decomposed into intermediate (1) and N_2_. Intermediate (1) was then mainly decomposed to product (7) and N_2_, with the accompanying production of intermediates (2) and (5). Intermediates (2) and (5) decomposed in reverse to intermediate (1). The triazole ring in intermediate (2) was gradually opened, accompanied by the stepwise generation of intermediates (3) and (4). Finally, intermediate (4) decomposed into the final products (8) and (9). The four C=N double bonds on the triazole ring in intermediate (5) were cleaved to form intermediate (6) and N_2_. Intermediate (6) eventually decomposed to product (7).

### 2.4. Combustion Measurements

**Figure 12:**
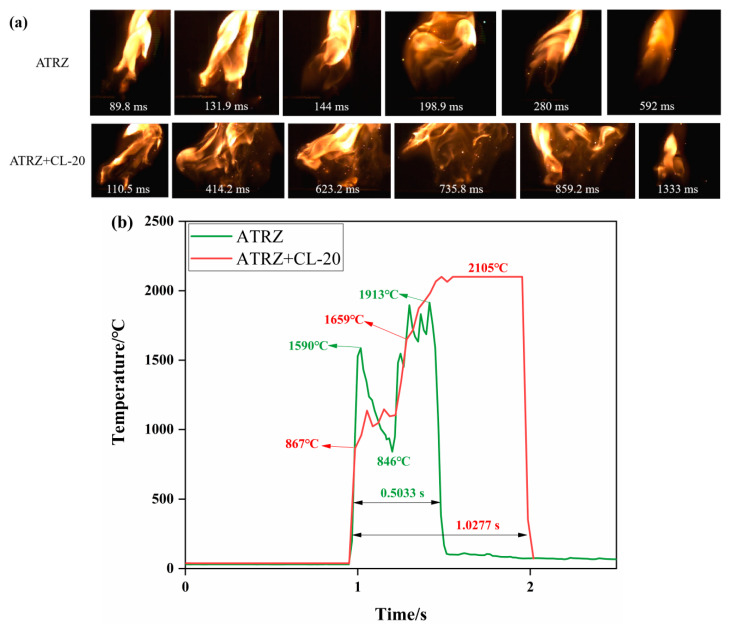
Combustion behaviors of ATRZ and ATRZ+CL-20 (**a**) and temperature evolution curves (**b**).

Figure 12a shows the combustion state of ATRZ and ATRZ+CL-20 burning in the air. The flame range of ATRZ+CL-20 was wider than that of ATRZ. Figure 12b shows the temperature change with time during the combustion of ATRZ and ATRZ+CL-20. ATRZ+CL-20 had a longer combustion duration and higher peak temperature than ATRZ. In the middle of the combustion process, ATRZ had an obvious temperature reduction stage, which may be the reason why the peak temperature of ATRZ was lower than that of ATRZ+CL-20. The combustion duration of ATRZ was 0.5033 s, while that of ATRZ+CL-20 was 1.0277 s. This shows that the rapid combustion energy release of ATRZ promoted the combustion energy release of CL-20.

## 3. Materials and Methods

ATRZ was prepared by Beijing Institute of Technology and had a purity of 97%. CL-20 was obtained from Liaoning Qing Yang Special Chemical Co., Ltd. (Liaoyang, China). ATRZ and CL-20 were dried in a vacuum oven at 50 °C for 48 h before use to avoid the influence of water on the test. ATRZ and CL-20 were evenly mixed using the mechanical mixing method at a mass ratio of 3:2.

**Figure 13:**
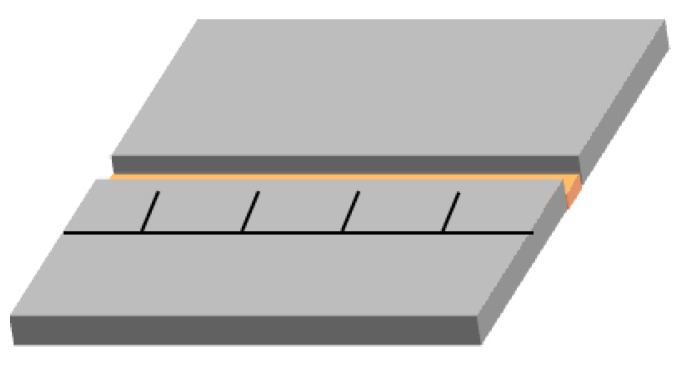
The schematic diagram of the groove.

A scanning electron microscope (SEM, S-4700 Hitachi, Tokyo, Japan) was used to explore the crystal morphology of the ATRZ, and the element distribution of the ATRZ was determined by an energy dispersive spectrometer (EDS) equipped on a SEM device. TG–DSC (STA 449F3, Netzsch, Serb, Germany) was used to analyze the thermal performance of ATRZ and CL-20. The TG–DSC test was carried out in an open crucible with an argon atmosphere and a 20 mL·min^−1^ gas flow rate, and the heating rates were 5 K·min^−1^, 10 K·min^−1^, 15 K·min^−1^, and 20 K·min^−1^, respectively. Coupling pyrolysis-gas chromatography mass spectrometry (PY-GC/MS) spectra were recorded by the EGA/PY-3030D apparatus and Shimadzu 2010 GC/MS apparatus (Shimadzu, Kyoto, Japan). About 2 mg of sample was placed in a quartz capillary tube of pyroprobe and the whole assembly was kept in the pyrolyzer for thermal decomposition at 400 °C for 12 s. The pyrolyzer was connected to gas chromatography. Helium was used as the carrier gas at a flow rate of 1 mL·min^−1^ with a back-up pressure of 10 psi. An Elite-5 capillary column (30 mm × 0.25 mm × 0.25 mm) was employed for the study, with cross-bonded diphenyl-5% and dimethyl polysiloxane-95% used as a stationary phase. Quadrupole mass spectrometer hyphenated with GC was used to record the mass spectra of the corresponding chromatogram. Afterwards, ~20 mg ATRZ and ATRZ+CL-20 were ignited by the CO_2_ laser with a power of 50 W and duration of 500 ms, and a highspeed camera (Qianyanlang X113, Hefei, China) was used to record the ignition and combustion processes at a speed of 10,000 fps. The sample was weighed for each experiment and uniformly placed in the groove. The schematic diagram of the groove is shown in Figure 13. The dimensions of the groove were 50.0 mm × 5.0 mm × 3.0 mm. The sample was pressed using a bar to ensure that its upper face was flat; the sample height was 2.00 mm.

## 4. Conclusions

The crystal morphology, non-isothermal thermal decomposition kinetics, and combustion behaviors of ATRZ are demonstrated in this work. The crystal morphology of ATRZ is an irregular long rod, and the length is about 150 μm. With the increase in the heating rate, the *T_p_* of ATRZ gradually increased, and the *T_p_* is stable at around 310 °C. Compared with CL-20, ATRZ has a higher thermal decomposition temperature. The thermal decomposition reaction of ATRZ involves the cleavage of the intermolecular N-N single bond and N=N double bond, and no intramolecular redox reaction occurs. The thermal decomposition of traditional ammonium nitrate explosives is mainly an intramolecular redox reaction, and the presence of nitro groups also greatly increases the sensitivity of CL-20. The E and lg*A* of ATRZ are higher than those of CL-20, which indicates that ATRZ has a better thermal stability and faster energy release rate. The pyrolysis path of ATRZ was investigated through a pyrolysis-gas chromatography mass spectrometry (PY-GC/MS) experiment. ATRZ has three different decomposition paths and finally generates N_2_, HC-N-CH, N≡C-N, and HC=N-C≡N. The rapid combustion energy release rate of ATRZ can stimulate and promote the combustion of CL-20. The combustion duration and flame temperature of ATRZ+CL-20 are significantly higher than those of ATRZ.

## Figures and Tables

**Table 1:** Peak temperatures and kinetics parameters of different pyrolysis systems obtained by Kissinger.

Samples	*β*/K·min^−1^	*T_p_* (°C)	*E_K_* (kJ/mol)	lg*A_K_* (s^−1^)
ATRZ	5	306.1	780.2	70.5
	10	309.3		
	15	310.1		
	20	310.9		
CL-20	5	235.4	239.3	24.3
	10	241.9		
	15	245.8		
	20	247.3		

**Table 2:** The functional expressions of 30 kinetic models *g*(*α*).

No.	*g*(*α*)
1	$\alpha^{2}$
2	α+(1−α)ln(1−α)
3	$(1-\frac{2}{3}\alpha )-{\left(1-\alpha \right)}^{\frac{2}{3}}$
4–5	${\left[1-{\left(1-\alpha \right)}^{\frac{1}{3}}\right]}^{n}(n=2,\frac{1}{2})$
6	${\left[1-{\left(1-\alpha \right)}^{\frac{1}{2}}\right]}^{\frac{1}{2}}$
7	${\left[{\left(1-\alpha \right)}^{\frac{1}{3}}-1\right]}^{2}$
8	${\left[1/{\left(1+\alpha \right)}^{\frac{1}{3}}-1\right]}^{2}$
9	−ln(1−α)
10–16	${\left[-\ln(1-\alpha )\right]}^{n}(n=\frac{2}{3},\frac{1}{2},\frac{1}{3},4,\frac{1}{4},2,3)$
17–22	$1-{\left(1-\alpha \right)}^{n}(n=\frac{1}{2},3,2,4,\frac{1}{3},\frac{1}{4})$
23–27	$\alpha^{n}(n=1,\frac{3}{2},\frac{1}{2},\frac{1}{3},\frac{1}{4})$
28	${\left(1-\alpha \right)}^{-1}$
29	${\left(1-\alpha \right)}^{-1}-1$
30	${\left(1-\alpha \right)}^{-\frac{1}{2}}$

**Table 3:** *E_a_* and lg*A* calculated by the three methods.

Methods	Ozawa	Satava–Sestak	Kissinger
*E_a_* (kJ/mol)	751.1	762.1	780.2
lg*A* (s^−1^)	71.8	71.1	70.5

## Data Availability

Request from the corresponding author of this article.

## Abbreviations

ATRZ	4,4′-azobis(1,2,4-triazole)
TG	Thermogravimetric
DSC	Differential scanning calorimetry
PY-GC/MS	Pyrolysis-gas chromatography mass spectrometry
*T_p_*	Peak temperature
*β*	Heating rate
*α*	Extent of conversion
*E_a_*	Activation energy
*E_K_*	Activation energy for the Kissinger method
*E_O_*	Activation energy for the Ozawa method
*E_S_*	Activation energy for the Satava–Sestak method
lg*A*	Pre-exponential factor
lg*A_K_*	Pre-exponential factor for the Kissinger method
lg*A_O_*	Pre-exponential factor for the Ozawa method
lg*A_S_*	Pre-exponential factor for the Satava–Sestak method
CL-20	2,4,6,8,10,12-hexanitro-2,4,6,8,10,12-hexaazaisowurtzitane
SEM	Scanning electron microscope
EDS	Energy dispersive spectrometer
